# First-Line Administration of Fibrinogen Concentrate in the Bleeding Trauma Patient: Searching for Effective Dosages and Optimal Post-Treatment Levels Limiting Massive Transfusion—Further Results of the RETIC Study

**DOI:** 10.3390/jcm10173930

**Published:** 2021-08-31

**Authors:** Nicole Innerhofer, Benjamin Treichl, Christopher Rugg, Dietmar Fries, Markus Mittermayr, Tobias Hell, Elgar Oswald, Petra Innerhofer, on behalf of the RETIC Study Group

**Affiliations:** 1 Department of Anesthesiology and Intensive Care Medicine, Medical University of Innsbruck, 6020 Innsbruck, Austria; benjamin.treichl@i-med.ac.at (B.T.); christopher.rugg@tirol-kliniken.at (C.R.); dietmar.fries@i-med.ac.at (D.F.); markus.mittermayr@i-med.ac.at (M.M.); elgar.oswald@tirol-kliniken.at (E.O.); petra.innerhofer@i-med.ac.at (P.I.); 2 Department of Mathematics, Faculty of Mathematics, Computer Science and Physics, University of Innsbruck, 6020 Innsbruck, Austria; tobias.hell@uibk.ac.at

**Keywords:** fibrinogen concentrate, fibrinogen plasma, massive transfusion, trauma induced coagulopathy, rotational thromboelastometry

## Abstract

Fibrinogen supplementation is recommended for treatment of severe trauma hemorrhage. However, required dosages and aimed for post-treatment fibrinogen levels remain a matter of discussion. Within the published RETIC study, adult patients suffering trauma-induced coagulopathy were randomly assigned to receive fibrinogen concentrate (FC) as first-line (*n* = 50) or crossover rescue (*n =* 20) therapy. Depending on bodyweight, a single dose of 3, 4, 5, or 6 g FC was administered and repeated if necessary (FibA10 < 9 mm). The dose-dependent response (changes in plasma fibrinogen and FibA10) was analyzed. Receiver operating characteristics (ROC) analysis regarding the need for massive transfusion and correlation analyses regarding fibrinogen concentrations and polymerization were performed. Median FC single doses amounted to 62.5 (57 to 66.66) mg·kg^−1^. One FC single-dose sufficiently corrected fibrinogen and FibA10 (median fibrinogen 213 mg·dL^−1^, median FibA10 11 mm) only in patients with baseline fibrinogen above 100 mg·dL^−1^ and FibA10 above 5 mm, repeated dosing was required in patients with lower baseline fibrinogen/FibA10. Fibrinogen increased by 83 or 107 mg·dL^−1^ and FibA10 by 4 or 4.5 mm after single or double dose of FC, respectively. ROC curve analysis revealed post-treatment fibrinogen levels under 204.5 mg·dL^−1^ to predict the need for massive transfusion (AUC 0.652; specificity: 0.667; sensitivity: 0.688). Baseline fibrinogen/FibA10 levels should be considered for FC dosing as only sufficiently corrected post-treatment levels limit transfusion requirements.

## 1. Introduction

During ongoing bleeding, fibrinogen is the first coagulation factor to reach critical thresholds [1]. On the one hand, blood loss, consumption, dilution, and fibrinolysis synergistically reduce fibrinogen levels, while on the other, missing fibrinogen storages and limited rapid hepatic synthesis fail to compensate. However, sufficient amounts of fibrinogen are of utmost importance for stable clot formation and cessation of bleeding. Several studies have shown that hypofibrinogenemia frequently occurs, particularly in young trauma patients, and that hypofibrinogenemia or poor fibrin polymerization at admission is associated with transfusion requirements and mortality [2,3,4,5,6].

The 5th Edition of the European Guidelines on Management of Major Bleeding and Coagulopathy Following Trauma recommend early and repeated monitoring of fibrinogen concentrations and/or polymerization and fast correction of deficiencies [7]. As an initial dose, 3 to 4 g fibrinogen concentrate (FC) or 15–20 single donor units of cryoprecipitate are recommended. However, the underlying evidence is based on experimental studies in pigs [8,9], retrospective studies and the use of FC in cardiovascular and liver surgery [10,11,12,13,14,15,16]. Up to date, prospective controlled studies in major trauma remain scarce [17,18,19]. In addition, recommendations on fibrinogen supplementation vary considerably among published guidelines on the management of severe bleeding [20].

Further knowledge on how fibrinogen levels and fibrin polymerization respond to first-line FC administration in the bleeding trauma patient is urgently needed. Post-treatment fibrinogen concentrations required to prevent ongoing of bleeding need to be evaluated. Last, comparative data on how fibrinogen concentration measurements correspond to common viscoelastic tests are substantial in the bleeding trauma patient receiving FC as both methods are used in clinical practice. In order to approach these issues, we here analyze data of patients within the RETIC study (“Reversal of Trauma-induced Coagulopathy using First-line Coagulation Factor Concentrates or Fresh-Frozen Plasma”) in which acute trauma patients (Injury Severity Score (ISS) > 15) with clinical signs of or a risk for substantial hemorrhage randomly received standardized body weight-dependent FC doses as first-line or rescue therapy if double-dose first-line FFP administration failed to correct coagulopathy [21].

## 2. Materials and Methods

### 2.1. The RETIC Study Procedure

The RETIC study, a single-center, parallel-group, open-labeled, randomized trial was approved by the Ethical Committee of the Medical University of Innsbruck, Austria (UN4497; October 2011; Chairperson P. Lukas) and registered with ClinTrials.gov (NCT01545635) and EudraCT (2011-004139-29) [21]. The Ethics Committee waived the need for initial informed consent, but written informed consent was obtained as soon as the patient regained legal capacity.

In brief, patients presenting with an Injury Severity Score (ISS) > 15, clinical signs or risk for substantial hemorrhage, and thromboelastometry-diagnosed poor fibrin polymerization (FibA10 < 9 mm) and/or prolonged initiation of coagulation (ExTEM CT > 90 s) randomly received up-rounded dosages of either FFP (at least 15 mL.kg^−1^) or coagulation factor concentrates (CFC), mainly FC (at least 50 mg·kg^−1^) as a single-dose. A 4-factor prothrombin complex concentrate and factor XIII concentrate were also administered in selected cases. Concerning tests also relevant for the presented study, bedside thromboelastometric assessment was performed with the ROTEM^®^ delta (ROTEM, TEM Innovations GmbH, Munich, Germany) and the according fluid reagents ExTEM and FibTEM. Further coagulation tests and blood count were performed at the Central Institute of Medical and Chemical Laboratory Diagnostics, University Hospitals of Innsbruck, Austria. For fibrinogen levels (Clauss method) the Multifibren^®^ U-assay (Siemens Healthcare Diagnostics, Marburg, Germany; reference range 210 to 400 mg·dL^−1^) was used. All used coagulation factor concentrates were produced by CSL Behring (Marburg, Germany). 

As the minimum amount of administered FC was to be 50 mg·kg^−1^ a dosing chart defining four body weight (BW) groups was applied to facilitate dose calculation (BW 45–50 kg: 3 g FC; 51–70 kg: 4 g FC; 71–100 kg: 5 g FC; >100 kg: 6 g FC). Before and 10 to 15 min after FC administration fibrinogen concentrations were measured and fibrin polymerization was assessed (FibA10, FibA30). The dosage was repeated if FibA10 remained below 9 mm or was borderline and diffuse or massive bleeding persisted. The same criteria indicated cross over rescue therapy initiation if applicable after double-dose study drug administration. All patients received one tranexamic acid bolus before the first study drug administration. Platelet concentrates were administered if clot firmness remained poor (ExTEM A10 < 35 mm) albeit sufficient fibrinogen polymerization (FibA10 > 15 mm) and/or to maintain platelets between 50 and 100 × 10^9^/L. Packed red blood cells (RBC) were administered to maintain hemoglobin levels between 80 and 100 g·L^−1^. The study protocol started with randomization and was continued until 24 h after ICU admission. According to the individual situation, patients received one or several treatment loops. Clinical data, details on fluid supply, and transfusion requirements were also collected until day 30 or hospital discharge. Massive transfusion was defined as transfusion of ≥10 RBC within 24 h.

The primary endpoint was to detect a difference in the occurrence of multiple organ failure (MOF) as assessed by SOFA score, in patients receiving first-line fresh-frozen plasma (FFP) or coagulation factor concentrates (CFC), mainly FC. 

Between March 2012 and February 2016, the RETIC study enrolled 100 patients with major trauma. Preplanned interim analysis after inclusion of 100 patients demanded early study termination as predefined stopping rules were met. Post randomization, six patients were excluded (erroneously inclusion, protocol deviation with loss of follow up), leaving data of 44 patients in the FFP group and 50 patients in the CFC group for final modified intent-to-treat analysis. First results have been previously published [21,22].

### 2.2. The Present Sub-Study

Within the RETIC study, 50 patients received FC as first-line and 23 patients as crossover rescue medication. 

We here include all patients with complete data sets (fibrinogen, FibA10, and FibA30) before and after FC administration. In the group of patients receiving FC as first-line therapy, complete data of all patients were available, among the 23 patients who received FC as rescue medication, 20 patients had a complete data set. Therefore, 70 patients were included.

As a predefined secondary outcome measure of the RETIC study protocol, we here analyze the response to first-line administration of standardized doses of FC during the first treatment loop. Post hoc we compare FC dosages and FibA30 changes with those expected by the previously described formula:

g FC = FibA30 change × body weight/140 [14].

Taking patients receiving FC as rescue medication into account as well, the potential of post-treatment fibrinogen levels to predict the need for massive transfusion will be evaluated and hence critical thresholds defined.

By using all in parallel performed measurements irrespective whether patients had received FC as first-line or rescue therapy, fibrinogen concentrations will be correlated to corresponding ROTEM (FibA10, FibA30) measurements.

### 2.3. Statistical Analysis

In this work, we performed preplanned descriptive and exploratory post hoc analyses. A mathematician not involved in the trial procedures or patient assessment (TH) conducted the statistical analyses using R, version 4.0.5. All statistical assessments were two-sided and a significance level of 5% was applied. Group differences were assessed by the Wilcoxon rank sum test for continuous variables and Fisher’s exact test for binary variables. Continuous data are presented as median (25th to 75th percentile) and categorical variables as frequencies (%). Effect size and precision are shown with estimated median differences between groups for continuous data and odds ratios (OR) for binary variables, with 95% confidence intervals (CIs). Logistic regression analysis for need of double dose FC predicted by baseline fibrinogen/FibA10 was performed. The estimated probability for double dose FC depending on baseline fibrinogen/FibA10 is presented with corresponding 95% CIs. ROC analysis was conducted for the binary outcome massive transfusion predicted by fibrinogen levels after completed FC supplementation during the first treatment loop providing AUC and a threshold for the fibrinogen level by maximizing Youden’s index. All completed pairs of measurements of fibrinogen concentration and fibrin polymerization (FibA10 and FibA30) during hemostatic resuscitation available in the RETIC trial were used to assess Pearson’s correlation for fibrinogen concentration, FibA10 and FibA30.

## 3. Results

Included patients’ characteristics and laboratory measurements at admission to emergency department are presented in Table 1.

**Figure 1:**
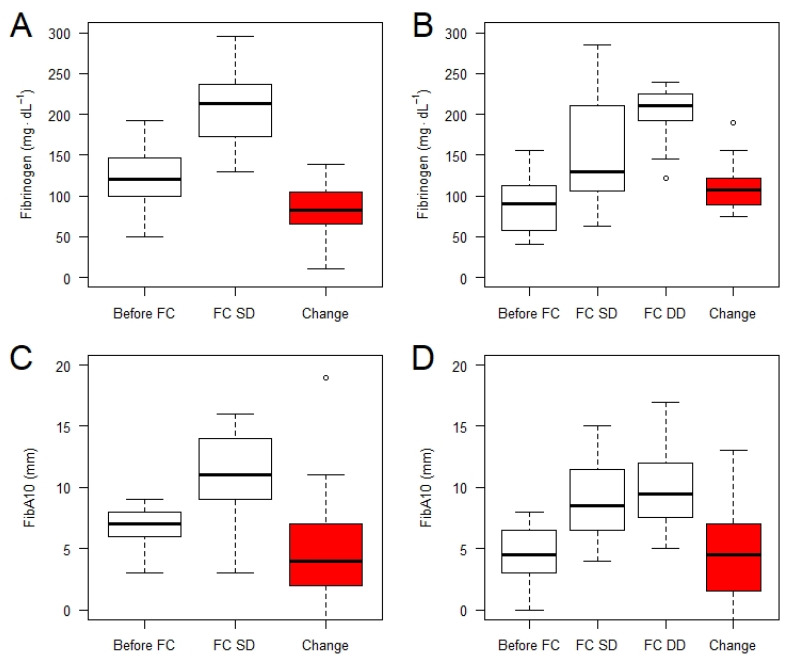
Change of fibrinogen concentration after supplementation with fibrinogen concentrate (FC) in patients successfully treated with a single dose (FC SD; *n* = 38) (**A**) and those who required a double dose (DD; *n* = 12) (**B**). The respective changes in fibrin polymerisation (Fibtem) are depicted in panel (**C**,**D**). Outliers are marked as °.

Regarding the 50 patients receiving FC as first-line therapy, the median age was 43 (27 to 51) years, body weight 80 (70 to 89) kg, ISS 35 (29 to 42), 76% were males, had median fibrinogen levels of 112 (93.5 to 141.5) mg·dL^−1^, and four patients exhibited hyperfibrinolysis. During the first treatment loop, 38 patients received a single dose of FC (5 (4 to 5) g) and 12 patients required a double dose (10 (8 to 10) g). Post hoc calculation of exact BW dependent FC dosages revealed that patients had received single doses of 62.5 (57 to 66.66) mg·kg^−1^ FC. Figure 1 depicts the response of fibrinogen and FibA10 values to single and double dose administration of FC. 

**Figure 2:**
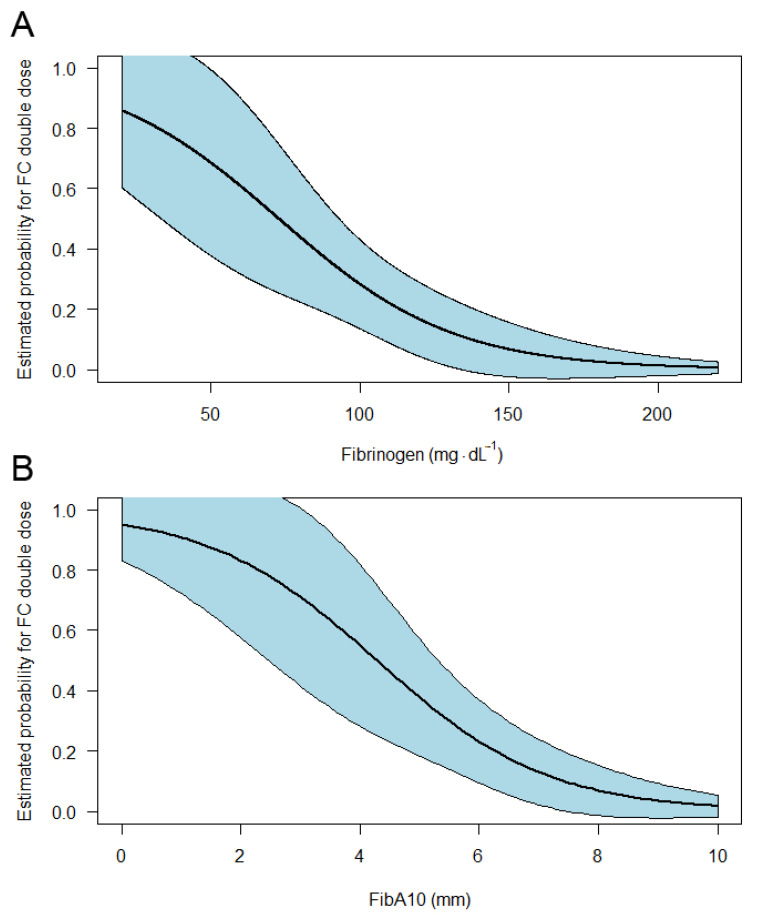
Estimated probability for the need of double dose fibrinogen concentrate administration in dependency of initial fibrinogen levels (**A**) and fibrin polymerization (FibA10, **B**).

Normalization of fibrinogen and/or FibA10 values was accomplished with a single dose of FC merely in patients with baseline fibrinogen above 100 mg·dL^−1^ and/or FibA10 above 5 mm (post-treatment: median fibrinogen 213 mg·dL^−1^, FibA10 11 mm). In patients with lower baseline values, double dose FC administration was required to achieve normal fibrinogen/FibA10 values. Median changes in fibrinogen concentrations were 83 mg·dL^−1^ or 107 mg·dL^−1^, in FibA10 values 4 mm or 4.5 mm after a single or double dose FC respectively. The estimated probability for the need of double dose FC with respect to baseline fibrinogen/FibA10 is illustrated in Figure 2.

Pairwise comparison of observed and formula expected [14] changes in fibrin polymerization after administration of a defined FC dosage showed that, in median 12.5 (4.83 to 20.63) mg·kg^−1^ more FC was required to achieve desired FibA30 changes (*p* = 0.0042). In our study, the FibA30 increased by 1 mm per 9.23 (7.32 to 14.29) mg·kg^−1^ FC—or per 0.74 (0.59–1.14) g FC in the “standard patient” with 80 kg BW.

**Figure 3:**
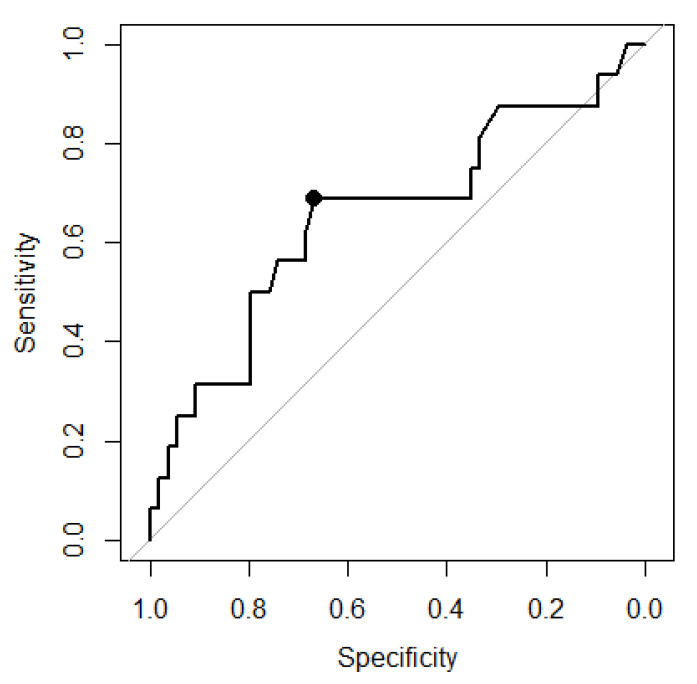
ROC analysis regarding post-treatment fibrinogen levels and the need for massive transfusion (AUC 0.6522). An optimal fibrinogen threshold (Youden index) of 204.5 mg·dL^−1^ was determined (specificity: 0.6667, sensitivity; 0.6875).

Moreover, considering the data of the 20 patients receiving FC as rescue medication, area under the ROC curve analysis revealed that fibrinogen levels after the first treatment loop significantly predicted the need for massive transfusion (MT) (AUC of 0.6521; Figure 3). 

Here, a threshold of 204.5 mg·dL^−1^ as assessed by Youden index maximization predicted MT with a specificity of 0.6666 and a sensitivity of 0.6875 (Figure 3). In general, 16 of 70 study patients (22.9%) were in need of MT; 11 of the 29 patients (37.9%) with post-treatment fibrinogen concentrations below and five of the 41 patients (12.2%) with post-treatment fibrinogen concentrations above the above-mentioned threshold (OR 4.35 (1.1627 to 20), *p* = 0.0194). Patients in need of MT were generally more severely injured and presented with lower hemoglobin and coagulation measurements at admission (data not shown). After the first treatment loop, patients with MT had similar Hb values (median 8.85 vs. 9.05 g·dL^−1^) but significantly lower platelet counts (median 61 vs. 124 g·L^−1^) when compared to patients without MT.

**Figure 4:**
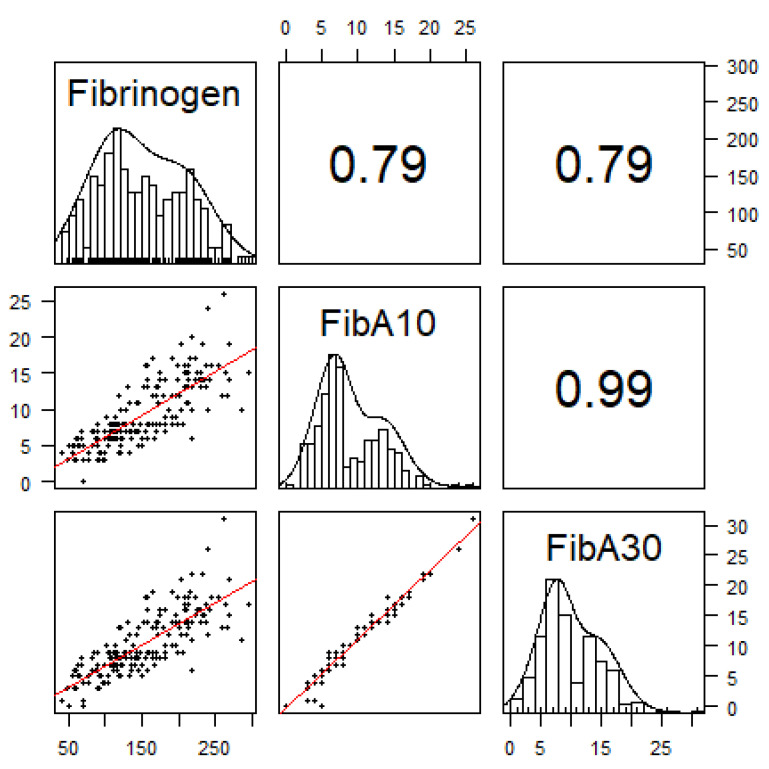
Pearson’s correlation: The panels show the distribution of fibrinogen measurements [Clauss’ method (mg·dL^−1^)] and its correlation to fibrin polymerization (mm) at 10 min (FibA10) and 30 min (FibA30) using all in parallel performed measurements (*n* = 193). Correlation coefficients were 0.79 between fibrinogen concentrations and FibA10 as well as FibA30 and 0.99 between FibA10 and FibA30.

Including all parallel performed measurements of fibrinogen concentration and fibrin polymerization (FibA10/FibA30) during hemostatic resuscitation (*n* = 193), a strong correlation of fibrinogen plasma concentration with FibA10 (r = 0.79) and FibA30 (r = 0.79) as well as between FibA10 and FibA30 (r = 0.99) was shown (Figure 4). 

The median difference between FibA30 and FibA10 was 1 (1 to 1) mm. Linear regression modeling revealed the following correlation between FibA10 and fibrinogen concentrations:

- FibA10 < 5 mm: fibrinogen concentrations < 100 mg·dL^−1^
- FibA10 = 8 mm (study intervention threshold): fibrinogen concentrations < 150 mg·dL^−1^
- FibA10 > 10 mm: fibrinogen concentrations > 150 mg·dL^−1^
- FibA10 > 14 mm: fibrinogen concentrations > 200 mg·dL^−1^.

## 4. Discussion

As a preplanned secondary endpoint of the RETIC study, the response of fibrinogen levels and fibrin polymerization to controlled first-line administration of FC during severe trauma related hemorrhage was analyzed. In patients with initial fibrinogen levels above 100 mg·dL^−1^ and FibA10 above 5 mm, a median single dose of 63 mg·kg^−1^ FC increased fibrinogen levels by 83 (66 to 138) mg·dL^−1^ and FibA10 by 4 (2 to 7) mm, hereby normalizing fibrinogen and FibA10 levels (median: 213 mg·dL^−1^ and 11 mm, respectively). In patients with lower baseline fibrinogen and FibA10 levels, normalization was merely achieved after administering a double dose of FC (median: 126 mg·dL^−1^). Here, the observed response was proportionally lower (median increase in fibrinogen levels: 107 mg·dL^−1^; in FibA10: 4.5 mm). The estimated probability of requiring a double-dose FC increased clearly with decreasing baseline fibrinogen/FibA10 values. Furthermore, post-treatment fibrinogen levels were shown to predict the need for massive transfusion.

The observed increase in fibrinogen levels (median: 83 mg·dL^−1^) after administration of a median of 5 g FC (63 mg·kg^−1^) is in line with results of two other prospective randomized trials investigating the feasibility of early FC administration [17,18]. In contrast to our study, both studies administered fixed and slightly higher doses of FC and treatment effect measurements were done at later time points. In the study by Curry and co-workers, 24 patients received 6 g FC leading to a mean increase in fibrinogen levels from 160 (70) to 280 (90) mg·dL^−1^ after two hours. The average change in fibrinogen levels were 90 (50) mg·dL^−1^ [17]. Similar results were reported by Nascimento and co-workers, where mean fibrinogen change was 93 mg·dL^−1^ and levels increased from 191 mg·dL^−1^ to 271 mg·dL^−1^ one hour after administration of 6 g FC in 21 patients [18]. In strong contrast to our and the above cited findings, a retrospective as well as a pharmacokinetic study has reported that the administration of 2 g FC (30 mg·kg^−1^) was able to increase fibrinogen levels by 100 mg·dL^−1^ [11,23]. Then again, these results have already been challenged in a work of Schlimp and co-workers, where neither fibrinogen nor FibA10 levels changed significantly from values at admission to ED in 85 trauma patients receiving a comparable low-dose of FC (2 to 3 g) [13]. In the FIinTIC study FibMCF increased from median 13 (11 to 15) mm to 15 (13.5 to 17) mm in patients of the verum group (*n* = 28) after prehospital administration of FC at dosages comparable to our study [19]. 

Seeebold et al. included 36 patients with an initial fibrinogen of 170 (130 to 220) mg·dL^−1^ and FibA5 of 6 (3.5 to 7) mm [24]. Approximately 30 min after receiving 4 g FC the FibA5 increased to 11 (8 to 12 mm). Taking total fibrinogen supplementation into account (FC, Cryoprecipitate and FFP) the authors calculated that per 1 g of administered fibrinogen, in median, FibA5 increased by 1 mm and fibrinogen levels by 20 mg·dL^−1^, respectively. 

In our study, FibA30 increased by 1 mm per 9.23 (7.32 to 14.29) mg·kg^−1^ FC. With regard to the “standard patient” with 80 kg BW, this translates to 1 mm FibA30 increase per 0.74 (0.59–1.14) g FC. FC requirements in this study were therefore higher than expected by a formula developed by two German University hospitals based on the experience with the management of massive bleeding [14]. They concluded that 6.25 mg·kg^−1^ or 0.56 g FC in the “standard patient” with 80 kg BW would increase FibA30 by 1 mm. Actually, a comparable response was observed in a retrospective study including 39 patients undergoing cardiac surgery. A 1 mm increase in FibA30 was achieved by 7.6 mg.-kg^−1^ FC [16]. However, initial fibrinogen levels were higher (190 mg·dL^−1^) than in our trauma patients and therefore, fibrinogen increased to levels above 300 mg·dL^−1^, and FibA30 increased by about 10 mm. One major reason for discrepant results may be the wrong assumption of a linear dose–response curve. Our data clearly show that, in the bleeding patient the response to FC depends on the severity of fibrinogen deficiency. In patients with initially lower fibrinogen/FibA10 values (fibrinogen < 100 mg·dL^−1^, FibA10 < 5 mm), 126 mg·kg^−1^ FC increased fibrinogen in median by 107 mg·dL^−1^ and FibA10 by 4.5 mm, whereas in patients with higher baseline values, half the dose (63 mg·kg^−1^) led to a median increase of 83 mg·dL^−1^ in fibrinogen levels and 4 mm in FibA10. Of course, those patients with severe fibrinogen deficiency also suffered more pronounced bleeding during the early phase of treatment as well. Furthermore, failure to promptly correct fibrinogen levels with the first single-dose FC certainly prolonged the phase of diffuse bleeding, then again counteracting with the intended increase of fibrinogen/FibA10. We truly believe that the response to FC supplementation would have been larger in those with severe fibrinogen deficiency if they had directly received a complete double dose FC (126 mg·kg^−1^) as initial dose. Thus, it seems more reliable to consider baseline values for determination of needed FC doses than to administer certain fixed doses, even if adjusted to BW. The dependency of the response to FC on baseline fibrinogen levels has not been described before and substantiates early fibrinogen supplementation at levels of 150 to 200 mg·dL^−1^ as recommended by most European Guidelines [20]. Awaiting lower thresholds not only increases FC doses required but also implies the acceptance of diminished efficacy regarding (i) post-treatment fibrinogen increase and (ii) the limitation of transfusion requirements.

Noteworthy, only few European guidelines even mention post-treatment goals for fibrinogen/FibA10 levels [20]. By ROC analysis, we found that post-treatment fibrinogen levels significantly predicted the need for massive transfusion. As optimal threshold, levels slightly above 200 mg·dL^−1^ corresponding to a FibA10 of 14 mm were determined. Interestingly, in children undergoing craniosynostosis surgery, associated with pronounced blood loss, early fibrinogen supplementation at a FibTEM threshold of <13 mm reduced blood loss and RBC requirements significantly when compared to supplementation at lower thresholds (FibTEM < 8 mm) [25]. In an in vitro study questioning the optimal level of fibrinogen, Bollinger and co-workers found that only fibrinogen levels above 200 mg·dL^−1^ enhance total clot formation [26]. Previously published results from the RETIC study confirmed that early achievement of fibrinogen levels above 200 mg·dL^−1^ not only normalized FibA10, but also total clot firmness, reduced the rate of MT significantly, and reduced platelet count decline and the need for platelet transfusion [21,22]. 

Considering all these findings, the efficacy of fibrinogen supplementation on limiting blood loss seems to strongly depend on timing and achievement of fibrinogen levels above 200 mg·dL^−1^, which is feasible when dosing FC according to initial fibrinogen levels.

With regard to diagnosing fibrinogen deficiency, although pure blood concentrations are certainly not always identical to functional polymerization measurements, both are used in clinical practice.

Obviously, the concentration of fibrinogen strongly influences its polymerization and fibrinolysis [27], however, so does thrombin generation, FXIII levels and the use of colloids, especially hydroxyethyl starches. In our study, hydroxyethyl starches were not in use, all patients received TXA and we maintained FXIII levels above 60% as FXIII supports fibrin polymerization and increases clot resistance to fibrinolytic attack. As summarized in a review [28] increased bleeding was reported in surgical patients with FXIII levels below 60% and at constant fibrinogen concentration fibrin polymerization declines below such FXIII levels. Observational data show that nearly 30% of trauma patients exhibit FXIII of less than 60% on admission, and its association with blood loss [29] FXIII levels is well maintained with FFP [30]. We thus aimed to maintain similar levels at about 60% in both groups in order to minimize further bias.

We here found a strong correlation between fibrinogen concentrations measured by Clauss’ method and the FibTEM assay, thereby confirming previously published data [6,31,32,33]. Furthermore, FibA10 values corresponded remarkably well to FibA30 values, validating its use as an early marker for therapeutic decision-making. 

The main limitations certainly include the open-labeled and single-center design of the study, albeit being prospectively conducted. However, the presented results were fairly comparable to those presented in two blinded, multicenter studies [17,18]. Furthermore, the study population was small, but up to date the largest population of trauma patients with prospectively collected fibrinogen/FibA10 measurements before and after each standardized FC dosage. In addition, our study enrolled a considerable proportion of patients with severe hypofibrinogenemia and for the first time reports an association of effective FC dosages and pretreatment fibrinogen levels. The need for MT clearly also depends on several other factors than fibrinogen alone, like severity of injury, pattern of injury, severity of blood loss before admission and so on. However, fibrinogen is at least a contributing factor and also one, which can be influenced by therapy. Moreover, early achievement of fibrinogen levels above 200 mg·dL^−1^ exerts a platelet saving effect and platelets further augment stable clot formation and limitation of blood loss [22].

## 5. Conclusions

The response to FC supplementation and moreover the body-weight-dependent dosage required to normalize fibrinogen/FibA10 values was shown to be dependent on baseline fibrinogen levels. As post-treatment fibrinogen concentrations also predict the need of MT, baseline adapted individual dosing may increase the efficacy of first-line FC administration in limiting blood loss.

## Figures and Tables

**Table 1:** Patient’s characteristics and laboratory measurements at admission to emergency department. Values are median (IQR) or numbers (%).

	Total (*n* = 70)
Age (ys)	43 (26 to 53)
Male sex (*n*)	49/70 (70%)
BMI (kg⋅m^−2^)	24.7 (22.9 to 26.2)
Time to ED (min)	60 (41 to 87)
ISS (pts)	35 (29–45)
Brain injury (*n*)	33/70 (47.1%)
AIS brain > 2 (*n*)	23/70 (32.9%)
GCS (pts)	12 (9–15)
Prehospital TXA (*n*)	6/70 (8.6%)
Intubation (*n*)	39/70 (55.7%)
Systolic BP (mm Hg)	101 (79 to 130)
Systolic BP < 90 mm Hg (*n*)	25/70 (35.7%)
Heart rate (bts⋅min^−1^)	105 (82 to 116)
pH	7.32 (7.24 to 7.36)
PH < 7.35 (*n*)	46/70 (65.7%)
BD (mmol·L^−1^)	4.45 (2.92 to 7.18)
BD < 6 (*n*)	50/70 (71.4%)
Hb (g·L^−1^)	112 (95 to 13)
PTI (%)	65.5 (51 to 76.5)
INR	1.3 (1.2 to 1.5)
aPTT (s)	33 (29 to 39.8)
Fibrinogen (mg·dL^−1^)	189 (128 to 219)
Platelets (G⋅L^−1^)	182 (153 to 213)
ExCT (s)	58 (51 to 69)
ExA10 (mm)	46 (39 to 50)
FibA10 (mm)	8 (5 to 10)

Binary data are presented as no./total no. (%), continuous data as medians (25th to 75th percentile). TXA, tranexamic acid; BD, base deficiency; PTI, prothrombintime index; ExCT and EX A10, extrinsically activated coagulation time and clot firmness at 10 min; FibA10, fibrin polymerization at 10 min.

## Data Availability

Because of privacy policy data cannot be shared.
